# Epidemiology and aetiology of sport‐related nasal fractures: Analysis of 599 Finnish patients

**DOI:** 10.1111/coa.13976

**Published:** 2022-09-09

**Authors:** Iida‐Kaisa Manninen, Tuomas Klockars, Laura K. Mäkinen, Karin Blomgren

**Affiliations:** ^1^ Department of Otorhinolaryngology—Head and Neck Surgery Helsinki University Hospital and University of Helsinki Helsinki Finland; ^2^ Department of Pulmonary Medicine, Heart and Lung Center Helsinki University Hospital and University of Helsinki Helsinki Finland; ^3^ HUS Joint Resources Helsinki University Hospital and University of Helsinki Helsinki Finland

**Keywords:** acquired nasal deformities, athletic injury, nasal bone, nasal fracture, nasal obstruction, sports injury

**Key Points**

1. Nasal fractures are related to a large number of different sports. Most nasal fractures occur in team sports (56%, 334/599).
2. Contact with another person is the most common injury mechanism for sport‐related nasal fracture (52%, 314/599).
3. Concomitant injuries are recorded in 14% (84/599) of patients and they are most prevalent in cycling (46%, 42/92) and equine sports (47%, 8/17).
4. Among popular team sports in the Helsinki area, basketball has the highest risk of nasal fracture.
5. Knowledge of the risks associated with different sports and the related injury mechanisms could help to better prevent nasal fractures.

## 1 INTRODUCTION

Sports activities cause 15% of nasal bone fractures.^1^ Nasal fractures are especially prevalent in team sports, such as basketball, soccer, American football, rugby and baseball, and in martial arts and equine sports.^1^, ^2^, ^3^ Similarly to other facial fractures, they are most prevalent among young males.^2^, ^4^

A nasal fracture and its treatment cause pain and decrease quality of life.^5^ The nose must be protected from impact for several weeks, causing a notable pause in training. Psychological and functional concerns regarding sports have been reported after nasal fracture treatment, which may be represented, for example, as an impact on sport performance, fear of reinjury or functional problems.^3^ Despite treatment, nasal fractures may later lead to impaired nasal breathing and aesthetic disadvantage.^6^

Our aim was to study the occurrence, characteristics and injury mechanisms of sport‐related nasal fractures.

## 2 MATERIALS AND METHODS

The STROBE reporting guidelines were used in manuscript preparation.

### 2.1 Design and participants

Individuals with nasal fracture were identified from the Helsinki University Hospital (HUH) electronic database for the years 2013–2018 using ICD‐10 codes S02.X, and from these individuals, patients with sport‐related nasal fractures were selected for further analyses. An inquiry was made to Finnish national sports federations to obtain the total number of athletes training in different team and combat sports.

### 2.2 Statistical analysis

Statistical analyses were performed together with an independent professional statistician. The data were analysed using IBM SPSS version 26 and NCSS 12 Statistical Software (2018). A *t*‐test was used to determine significant differences between means, Pearson's *χ* ^2^ to compare categorical variables, and crosstabs with pairwise *z*‐test with Bonferroni correction to analyse categorical variables between different sports groups. Statistical significance was set at *p* ≤ .05.

### 2.3 Ethical considerations

Institutional research permission was granted from HUH. No Research Ethics Board review was required.

## 3 RESULTS

### 3.1 Participants

Of the 5068 individuals with a facial fracture, 2465 (49%) had a nasal fracture, either exclusively or in combination with other fractures. One quarter (24%, 599/2465) of these were sport‐related.

**FIGURE 1:**
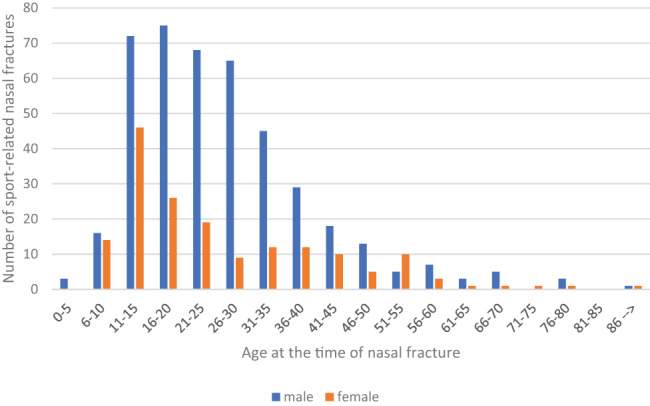
Age and sex distribution of 599 patients with sport‐related nasal fractures.

The mean age of patients with sport‐related nasal fracture was 26.3 years (y) (SD ± 14.2 y, median 23); no difference in mean ages between genders was noted (males, 26.3 y and females, 26.2 y; *p* = .29). Most (71%, 428/599) patients were male. One third (36%, 213/599) of patients were under 18 years old. Male dominance was less evident in non‐adult (62%, 133/213) patients than in adult (76%, 295/386) patients (*p* < .001; Figure 1).

### 3.2 Sports involved in nasal fractures

A total of 47 different sports activities were involved in nasal fracture cases (Table 1). Most of the nasal fractures occurred in team sports (56%, 334/599). The top seven sports were soccer (20.4%, 122/599), cycling (15.4%, 92/599), basketball (10.4%, 62/599), ice hockey (7.3%, 44/599), floorball (4.8%, 29/599), Finnish baseball (3.8%, 23/599) and boxing (3.8%, 23/599). Compared to adults, the proportion of non‐adult patients with nasal fractures was higher in team sports in general (62%, 133/213 vs. 52%, 201/386), in cheerleading (8%, 16/213 vs. 0.8%, 3/386), in Finnish baseball (6%, 13/213 vs. 3%, 10/386), in skateboarding (4%, 9/213 vs. 0.5%, 2/386), and in gymnastics (4%, 8/213 vs. 0.5%, 2/386) (*p* < .05). Adults had proportionally more nasal fractures caused by cycling (20%, 77/386 vs. 7%, 15/213), combat sports in general (15%, 57/386 vs. 5%, 11/213), wrestling (3%, 11/386 vs. 0% 0/213), and boxing (5%, 21/386 vs. 0.9%, 2/213) (*p* < .05). The ratio of nasal fractures occurring in different team and combat sports to the number of athletes participating in these sports is presented in Table 2.

**TABLE 1:** Distribution of 599 nasal fractures between sports, proportion of males, mean age of patients, and number of concomitant injuries

Sport	Frequency	Male proportion	Mean age	Concomitant injuries
	*N* (% of all cases)	*N* (% of males)		*N* (% of concomitant injuries associated with nasal fractures in the sport)
Team sports	334 (55.8)	257 (77)	23.5	20 (6)
Soccer	122 (20.4)	102 (84)	24.5	6 (5)
Basketball	62 (10.4)	42 (68)	21.8	0 (0)
Ice hockey	44 (7.3)	43 (98)	24.8	7 (16)
Floorball	29 (4.8)	27 (93)	25.5	1 (3)
Finnish baseball	23 (3.8)	15 (65)	22.0	4 (17)
Cheerleading	19 (3.2)	0 (0)	16.2	0 (0)
Handball	12 (2.0)	5 (42)	20.1	0 (0)
Rugby	7 (1.2)	7 (100)	26.7	1 (14)
Bandy	4 (0.7)	4 (100)	31.5	0 (0)
Cricket	3 (0.5)	3 (100)	25.3	0 (0)
American football	2 (0.3)	2 (100)	27.5	0 (0)
Rink bandy	2 (0.3)	2 (100)	40.0	0 (0)
Not recorded ball‐related sport	5 (0.8)	5 (100)	16.8	1 (20)
Individual sports	148 (24.7)	83 (56)	33.9	55 (37)
Cycling	92 (15.4)	58 (63)	39.4	42 (46)
Equine sports	17 (2.8)	1 (6)	33.9	8 (47)
Skateboarding	11 (1.8)	8 (73)	15.2	2 (18)
Swimming	8 (1.3)	3 (38)	29.8	0 (0)
Kick scooting	7 (1.2)	6 (86)	9.3	2 (29)
Downhill skiing	6 (1.0)	3 (50)	25.2	1 (17)
Surfing	3 (0.5)	2 (67)	24.7	0 (0)
Jogging	2 (0.3)	0 (0)	39.5	0 (0)
Snowboarding	2 (0.3)	2 (100)	20.0	0 (0)
Combat sports	68 (11.4)	58 (85)	26.5	5 (7)
Boxing	23 (3.8)	22 (96)	23.9	1 (4)
Wrestling	11 (1.8)	10 (91)	28.7	1 (9)
Thai boxing	8 (1.3)	7 (88)	27.9	1 (13)
Judo	7 (1.2)	6 (86)	22.4	0 (0)
Mixed martial arts	4 (0.7)	4 (100)	26.0	0 (0)
Jujitsu	3 (0.5)	2 (67)	24.3	0 (0)
Savate	2 (0.3)	2 (100)	37.5	0 (0)
Taekwondo	2 (0.3)	1 (50)	28.5	0 (0)
Karate	2 (0.3)	1 (50)	23.5	0 (0)
Taido	2 (0.3)	0 (0)	41.0	1 (50)
Not recorded combat sport	4 (0.7)	3 (75)	29.8	1 (25)
Others	30 (5.0)	16 (53)	17.6	1 (3)
Gymnastics	10 (1.7)	3 (30)	15.3	0 (0)
PE class^a^	9 (1.5)	7 (78)	12.9	0 (0)
Gym	5 (0.8)	3 (60)	29.8	1 (20)
Skating	3 (0.5)	2 (67)	11.7	0 (0)
Dance	3 (0.5)	1 (33)	25.3	0 (0)
Not recorded	7 (1.2)	7 (100)	27.1	0 (0)
Miscellaneous^b^	12 (2.0)	7 (58)	31.3	3 (25)
Total	599 (100.0)	428 (71)	26.3	84 (14)

^a^ Physical exercise class.

^b^ Only one case per sport.

**TABLE 2:** The ratio of nasal fractures reported over 1 year in the Helsinki University Hospital (HUH) district to the number of participating athletes per sport in the district (except where indicated) in the different team and combat sports with more than five nasal fractures in our study

Sport	Ratio to athletes
Rugby	1:196
Finnish baseball	1:407
Cheerleading	1:911
Wrestling	1:956^a^
Basketball	1:978
Boxing	1:983^a^
Handball	1:2050
Ice hockey	1:2210
Soccer	1:2505
Floorball	1:4418
Equine sport	1:5392

^a^ Ratio to athletes in Finland (population 5.5 million people). Sport federations did not know the number of athletes in the hospital district.

### 3.3 Injury mechanisms and concomitant injuries

Nasal fracture injury mechanisms are presented in Table 3. In cycling, the most common cause was a fall (76%, 70/92); 12% of cyclists had had a collision with a vehicle, 7% with another cyclist, and 5% with something else. Among team sports and combat sports, the situation in which a fracture happened was recorded in only 54% (219/402) of patient records; of these, competition was responsible for 65% (142/219) of the fractures and training for 35% (77/219).

**TABLE 3:** Injury mechanisms of 599 sport‐related nasal fractures

Injury mechanism	Frequency	Percent (%)
Contact with another person	**314**	**52**
Upper limb to nose	156	26
Elbow	112	19
Shoulder	12	2
Fist	5	0.8
Unspecified	27	4
Head to nose	54	9
Lower limb to nose	61	10
Knee	25	4
Shin	3	0.5
Heel	2	0.3
Unspecified	31	5
Other or unspecified	43	7
Contact with equipment	**96**	**16**
Ball	42	7
Stick	21	4
Puck	17	3
Other	19	3
Fall from a bike	**70**	**11**
Falling	**34**	**6**
Collision	**37**	**6**
Own knee hitting nose	**10**	**2**
Equine injury	**16**	**3**
Mounted	8	1
Unmounted	8	1
Unspecified	22	4

Concomitant injuries were recorded in 14% (84/599) of patients (Table 1). Concomitant injuries were more prevalent in cycling (46%, 42/92), equine sports (47%, 8/17), and individual sports in general (37%, 55/148), than in other sports (*p* < .05). Soccer (5%, 6/122), basketball (0%, 0/62), and team sports in general (6%, 20/334) carried a smaller risk of concomitant injury (*p* < .05). Concomitant injuries were more common among adults (17%, 64/386) than non‐adult patients (8%, 17/213) (*p* < .01). The most common concomitant injury was orbital fracture (5.2%, 31/599), followed by wound or superficial injury (2.8%, 17/599), tooth injury (2.3%, 14/599), Le Fort fracture (2.0%, 12/599) and intracranial injury (1.7%, 10/599).

## 4 DISCUSSION

### 4.1 Synopsis of key findings and comparison with other studies

Nasal fractures are related to large number of different sports (Table 1). With the exception of floorball and Finnish baseball, the sports distribution in our study resembles earlier findings.^1^, ^2^, ^3^, ^4^ Our study provides a comprehensive view of sport‐related nasal fractures, as practically all nasal fractures in our hospital district (population 1.6 million) are treated at HUH. To the best of our knowledge, this is the largest study on sport‐related nasal fractures.

Team sports, especially ball sports, and combat sports are overrepresented in sport‐related nasal fractures. Among popular team sports in the HUH district with more than 10 000 athletes (soccer, floorball, ice hockey and basketball), basketball had the highest risk of nasal fracture (Table 2). The combination of jumps, high speed and upward‐extended arms is a natural explanation for basketball‐related nasal fractures, as 77% (48/62) of them were caused by another player's upper limb.

Concomitant injuries were prevalent in sports with high‐energy injuries, such as cycling (46%) and equine sports (47%). Team sports carried a substantial risk of nasal fracture, but concomitant injuries were rare, which reflects lower injury energy. Injury energy also explains the higher prevalence of concomitant injuries among adults than among non‐adult patients.

Even though exercise at every level includes more training than competition, 65% of the nasal fractures among team and combat sports, in which the situation had been recorded, occurred during a game or match. This finding confirms previous observations.^2^, ^7^

Young males are most prone to sport‐related nasal fractures (Figure 1). In our study, 71% of patients were males, with a mean age of 26 years. Cannon et al. reported a similar gender distribution in patients with sport‐related nasal injuries, but patients in their study were clearly younger than ours (mean 18 y).^2^ Male dominance in sport‐related facial fractures has been more evident in a previous study (91% vs. 71%).^4^ The greater proportion of females in our study may be explained by gender equality in Finnish society, which encourages both sexes to practice a large variety of sports.

Sports caused one quarter (24%) of the nasal fractures in our study, which differs from the findings of a systematic review, reporting 15% of nasal fractures being sport‐related globally and 19% in Europe.^1^ The definition of “sport” and the low rates of traffic accidents in Finland, in part, explain these differences.

### 4.2 Clinical applicability

Nasal fractures may cause long‐term harm and also affect sports performance.^3^, ^6^ One quarter of patients with cosmetic rhinoplasty have a history of nasal fracture.^8^ Knowledge of injury mechanisms can help to prevent nasal fractures in the future (Table 3). Facial protection is already known to prevent facial injuries in ice hockey.^9^ Protective masks have been used among soccer players after nasal fracture to permit earlier return to sports activity.^10^ Helmets are already used in cycling, equine sports, boxing, and Finnish baseball. Modification to helmets, for example, with novel technology such as airbags, could be one option to reduce the risk of nasal fractures and protect the upper and middle face. Facial protection may be considered at least in cycling or equine sports, where concomitant injuries are prevalent. The problem in equine sports is that a notable proportion of the accidents occur while unmounted (Table 3). The use of protection only when riding is insufficient. Also, the presented information about the risk sports and injury mechanisms can be utilised when training pause is discussed with the athletes with nasal fracture.

### 4.3 Study limitations

Our study is based on patient records, which are always incomplete. Some aspects, such as the intensity of sports activities; the situation in which the injury occurred, for example competition or training; and recommended pause in sports training were poorly recorded. The number of athletes training different team and combat sports are estimates (Table 2). There are unregistered athletes training unorganised with their friends and their exact number is impossible to ascertain. Nevertheless, majority of the athletes training regularly are members of sport clubs and we considered the numbers between different team and combat sports comparable.

The definition of “sport” is ambiguous. Cycling is often reported separately from sport injuries. We defined “sport” as an activity combining physical effort and skill. Therefore, we included cycling without the influence of alcohol, but, for example, excluded walking and trampolining.

## 5 CONCLUSION

Nasal fractures are related to a large variety of sports. Young males are most prone to sport‐related nasal fractures, and team sports, especially ball sports, are overrepresented. Contact with another person is the most common injury mechanism (52%, 314/599). Nasal fractures may cause long‐term harm and also affect sports performance. Knowledge of the risks associated with different sports and the related injury mechanisms could help to better prevent nasal fractures.

## AUTHOR CONTRIBUTIONS

All authors participated in the study design. Iida‐Kaisa Manninen acquired and analysed the data. Iida‐Kaisa Manninen and Karin Blomgren drafted the first manuscript. Tuomas Klockars and Laura K. Mäkinen critically reviewed the manuscript. All authors participated in scientific discussion and approved the final manuscript.

## CONFLICT OF INTEREST

The authors declare no conflict of interest.

### PEER REVIEW

The peer review history for this article is available at https://publons.com/publon/10.1111/coa.13976.

## Data Availability

Research data are not shared but are available from the corresponding author upon reasonable request.
